# Molecular Characterization and Genomic Surveillance of SARS-CoV-2 Lineages in Central India

**DOI:** 10.3390/v16101608

**Published:** 2024-10-14

**Authors:** Purna Dwivedi, Mukul Sharma, Afzal Ansari, Arup Ghosh, Subasa C. Bishwal, Suman Kumar Ray, Manish Katiyar, Subbiah Kombiah, Ashok Kumar, Lalit Sahare, Mahendra Ukey, Pradip V. Barde, Aparup Das, Pushpendra Singh

**Affiliations:** 1 ICMR-National Institute of Research in Tribal Health, Jabalpur 482003, Madhya Pradesh, India; purna.dwivedi03@gmail.com (P.D.); smukul20@gmail.com (M.S.); md.afzal294@gmail.com (A.A.); arupneo2@gmail.com (A.G.); scbishwal@gmail.com (S.C.B.); dr.raysuman@gmail.com (S.K.R.); katiyarmanish10@gmail.com (M.K.); kombiahmaruthu@gmail.com (S.K.); vetashok5@gmail.com (A.K.); lalitkumarsahare@rediffmail.com (L.S.); mjukey@gmail.com (M.U.); aparupdas@nirth.res.in (A.D.); 2 Department of Microbiology and Biotechnology Centre, Faculty of Science, The Maharaja Sayajirao University of Baroda, Vadodara 390002, Gujarat, India; 3 Academy of Scientific and Innovative Research (AcSIR), Ghaziabad 201002, Uttar Pradesh, India

**Keywords:** SARS-CoV-2, genetic diversity, whole-genome sequencing, phylogenetic tree, COVID-19, transmission

## Abstract

Since the first reported case of COVID-19 in December 2019, several SARS-CoV-2 variants have evolved, and some of them have shown higher transmissibility, becoming the prevalent strains. Genomic epidemiological investigations into strains from different time points, including the early stages of the pandemic, are very crucial for understanding the evolution and transmission patterns. Using whole-genome sequences, our study describes the early landscape of SARS-CoV-2 variants in central India retrospectively (including the first known occurrence of SARS-CoV-2 in Madhya Pradesh). We performed amplicon-based whole-genome sequencing of randomly selected SARS-CoV-2 isolates (*n* = 38) collected between 2020 and 2022 at state level VRDL, ICMR-NIRTH, Jabalpur, from 11899 RT-qPCR-positive samples. We observed the presence of five lineages, namely B.1, B.1.1, B.1.36.8, B.1.195, and B.6, in 19 genomes from the first wave cases and variants of concern (VOCs) lineages, i.e., B.1.617.2 (Delta) and BA.2.10 (Omicron) in the second wave cases. There was a shift in mutational pattern in the spike protein coding region of SRAS-CoV-2 strains from the second wave in contrast to the first wave. In the first wave of infections, we observed variations in the ORF1Ab region, and with the emergence of Delta lineages, the D614G mutation associated with an increase in infectivity became a prominent change. We have identified five immune escape variants in the S gene, P681R, P681H, L452R, Q57H, and N501Y, in the isolates collected during the second wave. Furthermore, these genomes were compared with 2160 complete genome sequences reported from central India that encompass 109 different SARS-CoV-2 lineages. Among them, VOC lineages Delta (28.93%) and Omicron (56.11%) were circulating predominantly in this region. This study provides useful insights into the genetic diversity of SARS-CoV-2 strains over the initial course of the COVID-19 pandemic in central India.

## 1. Introduction

Coronavirus Disease 2019 (COVID-19) is caused by Severe Acute Respiratory Syndrome Coronavirus 2 (SARS-CoV-2). The first incidence of SARS-CoV-2 was reported from Wuhan City, China, in late December 2019, and the first genome sequence was submitted on 7 January 2020 (NCBI GenBank ID: MN908947.1) [1]. The World Health Organization declared COVID-19 a public health emergency on 30 January 2020 and declared it a pandemic on 11 March 2020 [2]. India too had witnessed the increased incidence of distinct SARS-CoV-2 strains in different states since the beginning of the pandemic [3]. By the end of 31 October 2023, it had been responsible for over 45 million cases and more than 0.53 million deaths in India [4]. SARS-CoV-2 was also found to show higher transmissibility compared to previously reported coronavirus outbreaks, i.e., SARS-CoV (2002) (WHO, 2003) and MERS-CoV (2012) [5]. On 27 January 2020, reports of the first COVID-19 case verified in India came from Kerala [6]. There are documented reports of various viral introductions brought about by international travelers. There were three waves of infections observed in India: the first wave occurred between 16 July 2020 and 31 January 2021, followed by the second wave from 16 March 2021 to 6 May 2021, and the third wave from 1 January 22 to 20 February 22 [7,8]. India, as one of the most populous countries with crowded cities, saw a surge in COVID-19 cases during March 2020. The virus also spread to rural areas and affected people of all age groups due to airborne transmission through viral particles and droplets, increased viral spread, and superspreading events linked to workers migrating from cities. In the state of Karnataka, the B.1.1.7 (Alpha) lineage was found to be predominant among many other lineages [9,10,11], and symptomatic cases have been linked to the pandemic’s early spread in the state. Three primary introductions of SARS-CoV-2 were identified in the state of Kerala by genomic sequencing and haplotype analysis [12]. These were followed by many outbreaks that caused the virus to spread locally [12]. In Gujarat state, lineages 20A and 20D were shown to be predominant among deceased patients with respiratory complications [13,14]. These lineages carry nucleotide changes C28854T in the N-gene and G25563T in Orf3a [13]. A collection of 200 genomes from the southern state of Telangana were sampled between March and July 2020 in order to assess the mutational landscape of SARS-CoV-2 isolates [14]. This analysis helped to detect mutations in non-structural proteins and demonstrated the preponderance of the 20B lineage [15]. Central India is a loosely defined geographical region of India, sometimes called *Madhya Bharat*, that consists of two Indian states: Madhya Pradesh (MP) and Chhattisgarh. Over 1.05 million cases and approximately 10,786 deaths were documented in this region as of 30 October 2023 [16]. Since the initial complete genome of SARS-CoV-2 from Wuhan was reported in early January 2020 [6], millions of whole-genome sequences worldwide have been added to public databases, i.e., GISAID and GENBANK, which have helped in the monitoring of SARS-CoV-2 variants. There has been a lack of genomic representation of SARS-CoV-2 from central India that needs to be addressed. Thus, more such studies representing different time frames and geographic regions are needed to understand the evolving landscape of the genetic diversity of the virus over the course of the pandemic. We have performed whole-genome sequencing of SARS-CoV-2 strains from the first and second waves of infections and performed comparative analysis with global strains to understand the dynamics of COVID-19.

## 2. Materials and Methods

### 2.1. Sample Acquisition

A total of 11,899 samples tested positive from 2.61 lakh tested cases for SARS-CoV-2 using real-time PCR (RT-PCR), and, among them, 38 samples underwent whole-genome sequencing and mutational analysis for the SARS-CoV-2 variants.

The Virus Research and Diagnostic Laboratory (VRDL) under the ICMR-National Institute of Research in Tribal Health, Jabalpur, is a designated state level (Grade-II) laboratory under the VRDL network established by the Department of Health Research, Ministry of Health & Family Welfare, Government of India. It was the first lab in Madhya Pradesh (central India) to start testing for suspected COVID-19 cases from Jabalpur and its surrounding districts of MP and Chhattisgarh. SARS-CoV-2-suspicious samples obtained from several quarantine camps and hospitals in Jabalpur area were processed for diagnostic testing in accordance with the WHO’s recommendations.

### 2.2. RNA Extraction and Real-Time Reverse Transcriptase PCR (RT-PCR)

During the period (March 2020 to May 2022), nasopharyngeal/oropharyngeal swabs (NPS/OPS) (*n*  =  263,124) were collected for routine SARS-CoV-2 diagnosis. A total of 140 μL of sample of the Virus Transport Medium (VTM) was used for viral RNA extraction using the QIAMP VIRAL RNA mini kit (Qiagen, Hilden, Germany). SARS-CoV-2 was confirmed using the ICMR-NIV multiplex single-tube SARS-CoV-2 RT-PCR assay kit by real-time RT-PCR. A total of 11,899 samples tested positive by real-time RT-PCR. Samples that displayed a cycle threshold (Ct) between 20 and 28 were considered eligible for sequencing to ensure maximum sequence coverage. Among positive samples, *n* = 19 samples from the very first reported cases of SARS-CoV-2 (Late March–April 2020) and that had international travel history were selected. Subsequently, *n* = 17 samples were randomly selected from the second wave, and *n* = 2 samples from the third wave were selected to see the presence of variants of concern (VOCs). The clinical and epidemiological details of the patients enrolled in the current study are shown in Table 1.

### 2.3. Library Preparation and Whole-Genome Sequencing

The cDNA was subjected to a multiplex PCR using the QIAseq SARS-CoV-2 primer panel (Qiagen GmbH, Germany). Subsequently, amplified products were purified using Agencourt AMPure XP beads (Beckman Coulter Inc., Brea, CA, USA) and quantified using the Qubit^TM^ dsDNA HS Assay Kit (Invitrogen, Waltham, Massachusetts, USA). The amplified cDNA sample was used for library preparation using the QIASeq FX DNA Library kit (Qiagen GmbH, Germany). The concentration and fragment size of the prepared libraries were checked using the Qubit Fluorometer (Thermofisher Scientific, Waltham, MA, USA) and Agilent Fragment Analyzer (Santa Clara, CA, USA) to make sure that the library profiles were in the expected range. The quality-checked normalized libraries were pooled and diluted to a final concentration of 10 pM, spiked with 5% PhiX. Whole-genome sequencing of first wave (*n* = 19) samples was performed on the Illumina iSeq platform using the iSeq 100 i1 Reagent v2 (San Diego, CA, USA) (300-cycle) kit, and subsequent samples of second and third waves were sequenced on the Ion GeneStudio S5 System platform using the Ion 540™ Chip Kit (Thermofisher Scientific, Waltham, MA, USA).

### 2.4. Variant Calling and Lineage Classification

The quality of the raw fastq files was assessed with the FastQC tool followed by the removal of low-quality bases, reads, and adapter sequencing using Trimmomatic [17,18]. The filtered sequence files were aligned to the SAS-CoV-2 reference genome (NC_045512.2), and reference-based assemblies were generated using BCFtools [19,20]. Sequence variations and lineage/clade classification were performed using Nextclade webserver followed by the identification of functional impacts [21]. The effect of the mutation on the structure of the protein was predicted using iStable [22].

### 2.5. Comparison with Global Strains

We have downloaded all the complete SARS-CoV-2 genomes (*n* = 2160) (Table S1) from central India for the phylogenetic analysis of SARS-CoV-2 genome sequences generated in this study, which were aligned with the strains reported from central India, using Augur Nextstrain’s phylodynamic pipeline toolkit “https://github.com/nextstrain/ncov (accessed on 10 May 2024)” [23,24]. Afterward, the maximum likelihood phylogenetic tree was constructed using the Augur tree implementation pipeline with IQ-TREE v1.6.1 [25] with the default parameters. The selected metadata information plotted in the time-resolved phylogenetic tree was constructed using TreeTime v0.81 [26], which was then annotated and visualized in the iTOL website [27].

Furthermore, the metadata of the genomes belonging to the seven lineages identified in this study were downloaded from the GISAID database until 31 March 2023 to check the transmission and circulation on a global scale.

**Table 1:** Epidemiological features of laboratory-confirmed COVID-19 cases.

Sample-ID	Age (In Years)	Gender	District of Residence	Travel History/Contact	Date of Sample Collection	Sample Type	Symptoms
ICMR-NIRTH-S1	46	M	JABALPUR	NA	27 March 2020	Nasopharyngeal swab	fever sore throat
ICMR-NIRTH-S2	48	M	JABALPUR	NA	27 March 2020	Oropharyngeal swab	fever
ICMR-NIRTH-S3	24	M	JABALPUR	Germany	20 March 2020	Nasopharyngeal swab	fever cough
ICMR-NIRTH-S4	59	M	JABALPUR	Dubai, UAE	20 March 2020	Nasopharyngeal swab	fever cough
ICMR-NIRTH-S5	45	F	JABALPUR	Dubai, UAE	20 March 2020	Nasopharyngeal swab	cough sore throat
ICMR-NIRTH-S6	22	F	JABALPUR	Dubai, UAE	20 March 2020	Nasopharyngeal swab	cough
ICMR-NIRTH-S7	53	M	JABALPUR	Dubai (contact of ICMR-NIRTH-S4)	21 March 2020	Nasopharyngeal swab	cough
ICMR-NIRTH-S8	40	M	JABALPUR	NA	22 March 2020	Nasopharyngeal swab	cough body ache
ICMR-NIRTH-S11	33	M	CHHINDWARA	Indore travel history	1 April 2020	Nasopharyngeal swab	fever cough breathlessness sore throat body ach
ICMR-NIRTH-S12	26	F	CHHINDWARA	Delhi travel history	7 April 2020	Nasopharyngeal swab	fever sore throat
ICMR-NIRTH-S13	27	M	CHHINDWARA	Contact of ICMR-NIRTH-S12	7 April 2020	Nasopharyngeal swab	cough sore throat
ICMR-NIRTH-S14	61	M	JABALPUR	Delhi travel history	8 April 2020	Nasopharyngeal swab	Fever cough breathlessness body ach sputum
ICMR-NIRTH-S15	30	M	INDORE	Indore travel history	10 April 2020	Nasopharyngeal swab	fever cough sore throat
ICMR-NIRTH-S16	22	M	INDORE	Indore travel history	10 April 2020	Nasopharyngeal swab	fever sore throat
ICMR-NIRTH-S17	24	M	SATNA	Indore travel history	10 April 2020	Nasopharyngeal swab	cough sore throat
ICMR-NIRTH-S18	70	M	JABALPUR	NA	11 April 2020	Nasopharyngeal swab	fever cough sore throat
ICMR-NIRTH-S19	50	M	JABALPUR	Contact of ICMR-NIRTH-S18	14 April 2020	Nasopharyngeal swab	fever sore throat
ICMR-NIRTH-S22	44	F	JABALPUR	Contact of ICMR-NIRTH-S19	17 April 2020	Throat swab	cough sore throat
ICMR-NIRTH-S23	35	M	BALAGHAT	NA	27 July 2020	Throat swab	Asymptomatic
ICMR-NIRTH-S24	28	F	BALAGHAT	NA	16 April 2021	Throat swab	Asymptomatic
ICMR-NIRTH-S25	28	M	BALAGHAT	NA	16 April 2021	Throat swab	Asymptomatic
ICMR-NIRTH-S26	24	M	BALAGHAT	NA	17 April 2021	Throat swab	Asymptomatic
ICMR-NIRTH-S27	24	M	BALAGHAT	NA	18 April 2021	Throat swab	Symptomatic
ICMR-NIRTH-S28	38	M	BALAGHAT	NA	19 April 2021	Throat swab	Symptomatic
ICMR-NIRTH-S29	25	M	BALAGHAT	NA	19 April 2021	Throat swab	Asymptomatic
ICMR-NIRTH-S30	19	M	BALAGHAT	NA	20 April 2021	Throat swab	Asymptomatic
ICMR-NIRTH-S31	14	M	BALAGHAT	NA	9 April 2021	Throat swab	Asymptomatic
ICMR-NIRTH-S32	45	M	BALAGHAT	NA	9 April 2021	Throat swab	Asymptomatic
ICMR-NIRTH-S33	70	F	BALAGHAT	NA	12 April 2021	Throat swab	Symptomatic
ICMR-NIRTH-S34	54	M	BALAGHAT	NA	13 April 2021	Throat swab	Asymptomatic
ICMR-NIRTH-S35	41	M	BALAGHAT	NA	18 April 2021	Throat swab	Asymptomatic
ICMR-NIRTH-S36	18	M	BALAGHAT	NA	21 April 2021	Throat swab	Asymptomatic
ICMR-NIRTH-S37	45	F	BALAGHAT	NA	24 April 2021	Throat swab	Asymptomatic
ICMR-NIRTH-S38	26	F	BALAGHAT	NA	3 February 2022	Throat swab	Asymptomatic
ICMR-NIRTH-S39	40	F	BALAGHAT	NA	4 February 2022	Throat swab	Asymptomatic
ICMR-NIRTH-S40	40	M	BALAGHAT	NA	20 April 2021	Throat swab	Asymptomatic
ICMR-NIRTH-S41	25	M	BALAGHAT	NA	21 April 2021	Throat swab	Asymptomatic
ICMR-NIRTH-S42	16	F	BALAGHAT	NA	27 April 2021	Throat swab	Asymptomatic

NA = contact history not available.

## 3. Results

### 3.1. Genome Assembly, Lineage Assignment, and Mutational Analysis

All these SARS-CoV-2 whole-genome sequences mapped to the reference strain (Wuhan/Hu-1/2019, NC_045512.3) with 99.1–99.8% identity. The consensus assembly of 38 samples has been deposited in the BioProject ID: PRJNA759056 (Table S2). To analyze the mutation pattern, these 38 genomes were analyzed with the Auspice visualization tool of Nextclade [21] (Figure S1). Nextstrain clades and the Pangolin classification system were used for classifying the phylogenetic lineages, and their corresponding GISAID clade is shown in Table 2. These 38 SARS-CoV-2 strains belong to seven lineages, namely B.1.617 (Delta) (*n* = 17), B.1.195 (*n* = 8), B.1 (*n* = 6), B.1.36.8 (*n* = 2), BA.2.10 (Omicron) (*n* = 2), B.1.1 (*n* = 2), and B.6 (*n* = 1). This revealed *n* = 186 single nucleotide polymorphisms (*n* = 129 non-synonymous and *n* = 57 synonymous changes), as shown in Table S3. Even though approx. half of these samples are from the early stage of the pandemic, it is interesting to note that only one strain (ICMR-NIRTH-S14, lineage B.6, clade O (19A), date of collection: 8 April 2020) did not show a well-characterized D614G mutation in spike protein (Table 2).

In total, 29 novel nucleotide variations in the sequences of the SARS-CoV-2 genome have been identified from 38 patient isolates which were unique to the sample and not found in the genomes reported from the central India state, as per the data retrieved from the GISAID database. In the present study, 17 patients were asymptomatic, while the remaining 21 had symptoms. Upon comparing the distribution of the 29 novel mutations among our study samples, we found that a few mutations such as A4533G, A4778T, and G6167T were present only among the symptomatic ones (Tables S3 and S4).

### 3.2. Effect of Novel Mutations on Protein Stability

Using an integrated sequence and structure predictor, iStable [22], the effects of the novel 29 mutations identified were determined. Among 29 mutations, 20 mutations were only identified in the ORF1ab region, which was known to encompass mutational spectra. Out of these 29 mutations, 17 mutations were non-synonymous, 11 were synonymous, and 1 mutation was found in the non-coding region. A total of 15 non-synonymous mutations were found to confer deleterious effects on protein function as they scored in a range between 0 and 1 (Table S4). As most of the mutations were found in ORF1ab region, it was interesting to note that the mutational spectra of this region should be considered while designing new antiviral therapeutics targeting viral ORF1ab.

### 3.3. Phylogenetic Analysis

**Figure 1:**
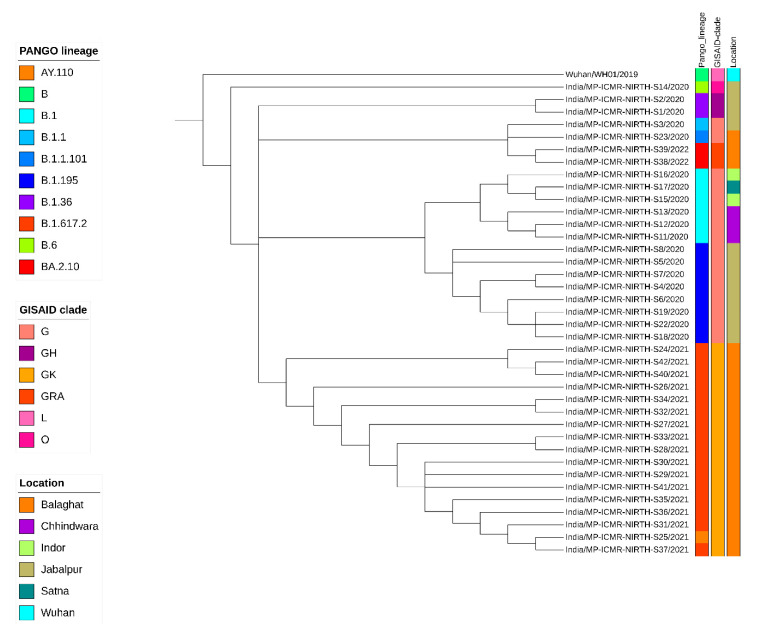
Phylogenetic analysis of the 38 SARS-CoV-2 genomes sequenced at ICMR-NIRTH with the reference genome (NC_045512) hCoV19/Wuhan/WH01/2019. Lineage distribution is depicted by different colors.

**Figure 2:**
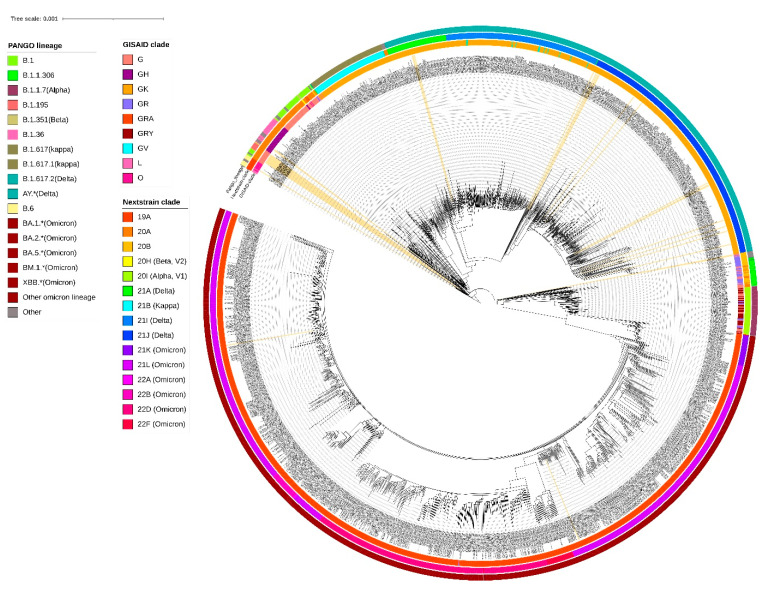
Phylogenetic analysis of the 38 SARS-CoV-2 genomes sequenced at ICMR-NIRTH with the 2160 genomes obtained from GISAID. Classification of the genome sequences according to the Pangolin lineages, Nextstrain, and GISAID clade is shown in color. The labels corresponding to the ICMR-NIRTH genome sequences generated during this study are highlighted and have been marked with light yellow color.

A neighbor-joining tree was constructed using *n* = 38 strains sequenced in this study (Figure 1), and their phylogenetic relationship, along with 2160 SARS-CoV-2, is shown in Figure 2, where the genomes sequenced in this study are shown in beige highlight. All the SARS-CoV-2 genomes reported so far from central India comprise 109 different SARS-CoV-2 Pangolin lineages. Importantly, these include B.1.1.7 (Alpha) (64/2160 = 2.96%), B.1.351 (Beta) (2/2160 = 0.09%), B.1.617.2 + AY.* (Delta) (625/2160 = 28.93%), and BA.* + BM.1.* + XBB.* (Omicron) (1212/2160 = 56.11%) VOCs as per the data submitted in GISAID until 31 March 2023 from central India.

A cluster of eight samples belonging to B.1.195 lineage (Figure 1) comprise three of the first four cases in Jabalpur (a family of three patients who had visited Dubai and tested positive on 20 March 2020) and their one contact (ICMR-NIRTH-S7, tested positive on 21 March 2020). The other three individuals in this cluster tested positive between 11 and 17 April 2020 and were also linked to each other. Another sample with a foreign travel history was ICMR-NIRTH-S3 (lineage B.1.1), who had returned from Germany and tested positive on 20 March 2020. We have analyzed this cluster, but this sample did not group with other strains reported from central India (Figure 2).

Another cluster represented B.1 lineage strains (*n* = 6 cases: three each from Chhindwara and Indore districts, which were epidemiologically linked) (Table 1). Two cases (ICMR-NIRTH-S1 and ICMR-NIRTH-S2) belonged to B.1.36.8 and had tested positive on the same day (27 March 2020). Sample ICMR-NIRTH-S14 (lineage B.6) belongs to a person who had traveled to Delhi and clusters together with other strains sequenced by the Defense Research and Development Establishment (DRDE), Gwalior (Figure 2).

The overall lineages distribution highlighted the dominant occurrence of B.1, B.1.36, and B.6 during the first wave, B.1.617.2 in the second, and omicron in the third wave in central India.

### 3.4. Distribution of Circulating Lineages

**Figure 3:**
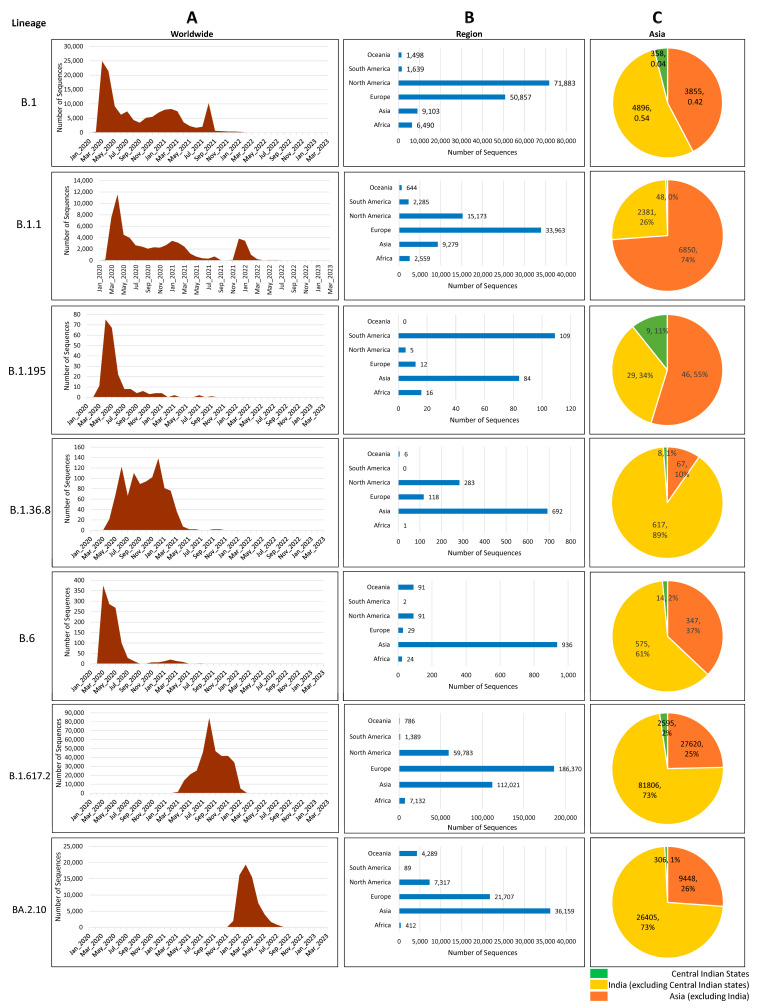
Global distribution of 5 lineages of SARS-CoV-2 from GISAID database until August 2022. (**A**) Month-wise distribution of lineages from worldwide submitted data. (**B**) Distribution of lineages across different continents of the world. (**C**) Comparison of lineages present in central Indian states with other Indian states and Asian countries.

We have investigated the global prevalence of the seven lineages by analyzing the genomes from GISAID and found that, together, North America and Europe shared nearly 86% of the load of B.1 and 83% of that of B.1.1; Europe and Asia shared nearly 82% of the load of Delta and Omicron VOCs. Though only 6.43% of the genomes of B.1 were observed in Asia, among them, nearly half of (3.7%) the genomes were submitted from India, indicating the transmission of B.1 in this region. Interestingly, strains belonging to lineage B.1.195 have not been observed after 2021, whereas lineages B1 and B.1.1 have been reported until very recently in the year 2022. In fact, in central India, B.1.1 was present in the year 2022, indicating that these strains were in circulation after the first report. B.1.36 was present in a small percentage during the first wave but surged during the second wave. Lineages B.6 and B.1.195, however, were prevalent in the first wave, and an almost negligible number of genomes were reported after the first wave. Together, South America and Asia have submitted about 85.3% of lineage B.1.195 so far. Furthermore, Asia alone submitted 62.9% of lineage B.1.36.8 and 79.7% of B.6 SARS-CoV-2 sequences, with most of the sequences coming from India (Figure 3).

## 4. Discussion

Since the first case of SARS-CoV-2 was discovered in the state of Kerala, India has been on alert for the spread of airborne viruses. To contain and stop the spread of viral transmission, the Ministry of Home Affairs (MHAs) subsequently declared a nationwide lockdown that would last from 25 March 2020 to 14 April 2020. A single case regarding lineage B.1.195 (associated with migration) identified in this study may indicate that the initial period (March–April 2020) had effective containment measures, especially for those who had returned from abroad. This strain was associated with travel history to Dubai and was also closely related to other patients from Gujarat and Brazil. All these individuals had a travel history to Dubai around a similar time. However, the subsequent introduction of several lineages via interstate travel might have been the major driver of SARS-CoV-2 transmission in central India. This is supported by the fact that a significant genomic diversity was found during the initial WGS analyses of SARS-CoV-2 patients who traveled to India from other countries.

Another observation from the genomic analysis carried out in this study was that the earliest documented evidence of Delta variants in MP (GISAID ID: EPI_ISL_2461258, Lineage: AY.122) was in September 2020. However, the proportion of this variant among the newly detected strains after May 2021 has increased to over 90% in a short span of time. This indicates its selective advantage over the other circulating lineages [28]. Delta and Omicron variants have been reported to be more transmissible than the other variants, and strong evidence is available which states that they were behind the emergence of the second and third waves of COVID-19 in central India [29,30]. These observations suggested that the diverse genotypes of SARS-CoV-2 may have emerged as a process of convergent evolution. In addition, certain mutations may lead to changes in their surface antigenic structure, whereby the antibodies due to previous infection/vaccination might not bind to them as effectively, exerting an ‘immune evasion’ advantage to them which can serve as a selection pressure and might be associated with ‘breakthrough infections’.

The D614G (37/38 strains) mutation has been shown to enhance the replication rate and transmissibility of SARS-CoV-2 by the more efficient binding of SARS-CoV-2 spike protein to human angiotensin converting enzyme-2 (ACE2) receptors and subsequently increases viral entrance into host cells. Mutations in the spike can enhance viral transmission, disease severity, and the virus’s capacity to elude immune defensive responses [31].

Five immune escape mutations in the S gene at a codon position other than D614G were L452R, P681R/H, Q57H, E484A, and N501Y during the second and third waves. Substitution at positions P681 and E484 has become increasingly common among clinical isolates. Previous studies showed that the virulence and pathogenesis of the Delta variant could be impacted by D614G and P681R mutations [32]. Aside from D614G, the B.1.617.2 lineage, which has the spike protein mutations L452R and N501Y, may explain the transmission and surge in cases in central India in March 2021. Although the D614G mutation occurs in the Delta and Omicron variants, the additional presence of the P681H mutation may result in slow cleavage. Moreover, this may limit the Omicron virus replication to the upper respiratory tract, resulting in less fusion and infectivity compared to the Delta and D614G  +  P681R double mutants. A previous study reported that 90.5% of the samples had the D614G mutation, which has recently been linked to higher viral loads and accelerated replication on human lung epithelial cells [33]. Among the S protein mutations, S: L452R in the receptor-binding domain (RBD) has also been associated with vaccine breakthrough infections by decreasing the neutralization capability of vaccine-induced immune response [33,34]. Another mutation S:E484Q in the Spike RBD has also been associated with a similar clinical impact but was missing in the cases we sequenced [34,35]. Studies tracking the selection of such mutations using intra-host variation suggest that there was an early indication of Spike protein mutation playing a key role in increased virulence and transmissibility among the population [36].

Among 29 mutations, 20 mutations were only identified in the ORF1ab region, which was known to encompass mutational spectra. ORF1ab is a conserved area in the SARS-CoV-2 genome [37]. Out of these 29 mutations, 17 mutations were non-synonymous, 11 were synonymous, and 1 mutation was found in the non-coding region. By improving the virus’s adaptability to the utilization of human codons, synonymous SARS-CoV-2 mutations associated with the action of various mutational mechanisms may have a positive effect on viral evolution [37]. The non-synonymous mutation causes epitope loss, which might be connected to immune evasion, which would increase viral pathogenicity and propagation [38].

However, after the emergence of new Omicron sub-lineage BA.5, it is now unknown if SARS-CoV-2 is shifting to a more gradual adaptive mechanism or if it will continue to evolve in this beneficial fashion with the frequent emergence of highly divergent lineages [39,40].

In 2022, multiple lineages emerging within BA.2, BA.5, and Omicron were observed in a more step-wise fashion, with several amino acid changes and moderate transmission advantages, which could indicate a shift to a more gradual stepwise evolution. The formation of VOCs and possible future antigenically different lineages may be viewed as ‘shift-like events’, which are unanticipated, major alterations in the virus’ genetic make-up and, possibly, therapeutically relevant features [39,40]. It is impossible to foresee where future significant lineages will emerge from in viral genetic diversity and whether they will arise from ‘shift-like’ or more gradual ‘drift-like’ progression similar to that seen in the Omicron clade during 2022 [40,41].

However, these limitations do not affect our conclusions about the genetic diversity and phylogenetic relatedness of the strains from central India. Overall, our study provides useful insights into the extent of the genetic diversity of SARS-CoV-2 strains over the course of the COVID-19 pandemic right from the very beginning to know its origin, dissemination, and evolving pattern of genetic diversity.

### Limitations of This Study

This study has a few limitations: (i) We have sequenced only a limited number of samples from central India. (ii) Although we identified new mutations in SARS-CoV-2 genomes, we were unable to link them with phenotypic effects. Despite these limitations, our study provides valuable insights into the genetic diversity of SARS-CoV-2 infections in central India and its trajectory through the two waves of the SARS-CoV-2 pandemic.

## 5. Conclusions

To our knowledge, this is among the very few studies in which the molecular surveillance-based phylogenetic trends based on whole-genome sequencing of SARS-CoV-2 has been undertaken from central India. In this study, we have performed WGS of 38 strains from Jabalpur and adjoining districts and compared them with over 2000 strains from various districts from central India representing different lineages. This study shows that the primary sources of COVID-19 introduction were those with a recent history of foreign travel in the affected countries. This was later on further amplified by interstate migration, which subsequently led to the emergence of newer strains.

The phylogenetic analysis of the SARS-CoV-2 strains in this study points to multiple introductions of the SARS-CoV-2 virus in central India. The genetic diversity, transmission, and evolution of SARS-CoV-2 exhibited a consistent pattern of increasing divergence within major lineages. Continued genomic surveillance strategies are needed for improved understanding of the SARS-CoV-2 pandemic. Furthermore, increased sequencing capacity is necessary for contact tracing and quicker identification of the transmission hotspots. This can be helpful for better preparedness, effective interventions, and surveillance activity.

The COVID-19 phenomena show that continuous and sustained monitoring of pathogens using next-generation sequencing is a useful tool for monitoring disease transmission and evolution patterns. The pandemic accelerated the capacity building at district level to enable molecular diagnostic with the capability of novel pathogen identification.

## Figures and Tables

**Table 2:** Pangolin lineage, GISAID clade, and Nextstrain clade information of the 38 SARS-CoV-2 genomes sequenced in this study.

Sr No	Sample ID	Date of Sample Collection	GISAID Clade	Nextstrain Clade	Pangolin Lineage	WHO Name
1	ICMR-NIRTH-S1	27 March 2020	GH	20A	B.1.36.8	unassigned
2	ICMR-NIRTH-S2	27 March 2020	GH	20A	B.1.36.8	unassigned
3	ICMR-NIRTH-S3	20 March 2020	G	20B	B.1.1	unassigned
4	ICMR-NIRTH-S4	20 March 2020	G	20A	B.1.195	unassigned
5	ICMR-NIRTH-S5	20 March 2020	G	20A	B.1.195	unassigned
6	ICMR-NIRTH-S6	20 March 2020	G	20A	B.1.195	unassigned
7	ICMR-NIRTH-S7	21 March 2020	G	20A	B.1.195	unassigned
8	ICMR-NIRTH-S8	22 March 2020	G	20A	B.1.195	unassigned
9	ICMR-NIRTH-S11	1 April 2020	G	20A	B.1	unassigned
10	ICMR-NIRTH-S12	7 April 2020	G	20A	B.1	unassigned
11	ICMR-NIRTH-S13	7 April 2020	G	20A	B.1	unassigned
12	ICMR-NIRTH-S14	8 April 2020	O	19A	B.6	unassigned
13	ICMR-NIRTH-S15	10 April 2020	G	20A	B.1	unassigned
14	ICMR-NIRTH-S16	10 April 2020	G	20A	B.1	unassigned
15	ICMR-NIRTH-S17	10 April 2020	G	20A	B.1	unassigned
16	ICMR-NIRTH-S18	11 April 2020	G	20A	B.1.195	unassigned
17	ICMR-NIRTH-S19	14 April 2020	G	20A	B.1.195	unassigned
18	ICMR-NIRTH-S22	17 April 2020	G	20A	B.1.195	unassigned
19	ICMR-NIRTH-S23	27 July 2020	G	20B	B.1.1.101	unassigned
20	ICMR-NIRTH-S24	16 April 2021	GK	21A	B.1.617.2	Delta
21	ICMR-NIRTH-S25	16 April 2021	GK	21J	B.1.617.2	Delta
22	ICMR-NIRTH-S26	17 April 2021	GK	21J	B.1.617.2	Delta
23	ICMR-NIRTH-S27	18 April 2021	GK	21J	B.1.617.2	Delta
24	ICMR-NIRTH-S28	19 April 2021	GK	21J	B.1.617.2	Delta
25	ICMR-NIRTH-S29	19 April 2021	GK	21J	B.1.617.2	Delta
26	ICMR-NIRTH-S30	20 April 2021	GK	21J	B.1.617.2	Delta
27	ICMR-NIRTH-S31	9 April 2021	GK	21J	B.1.617.2	Delta
28	ICMR-NIRTH-S32	9 April 2021	GK	21J	B.1.617.2	Delta
29	ICMR-NIRTH-S33	12 April 2021	GK	21J	B.1.617.2	Delta
30	ICMR-NIRTH-S34	13 April 2021	GK	21J	B.1.617.2	Delta
31	ICMR-NIRTH-S35	18 April 2021	GK	21J	B.1.617.2	Delta
32	ICMR-NIRTH-S36	21 April 2021	GK	21J	B.1.617.2	Delta
33	ICMR-NIRTH-S37	24 April 2021	GK	21J	B.1.617.2	Delta
34	ICMR-NIRTH-S38	3 February 2022	GRA	21L	BA.2.10	Omicron
35	ICMR-NIRTH-S39	4 February 2022	GRA	21L	BA.2.10	Omicron
36	ICMR-NIRTH-S40	20 April 2021	GK	21A	B.1.617.2	Delta
37	ICMR-NIRTH-S41	21 April 2021	GK	21J	B.1.617.2	Delta
38	ICMR-NIRTH-S42	27 April 2021	GK	21A	B.1.617.2	Delta

## Data Availability

All data generated or analyzed during this study are included in this article. The raw data used to support the findings of this study have been deposited in the NCBI with BioProject ID: PRJNA759056.

## Supplementary Materials

The following supporting information can be downloaded at: https://www.mdpi.com/article/10.3390/v16101608/s1, Figure S1: Genomic map showing coverage of the 38 SARS-CoV-2 genomes sequenced in this study; Table S1: Metadata file with information about the SARS-CoV-2 genome sequences from Central India available in GISAID database till 31 March 2023; Table S2: Genome assembly features of the SARS-CoV-2 genomes sequenced in this study; Table S3: Nextclade results of SARS-CoV-2 classification of the 38 genomes sequenced in this study along with the mutational profile of each strain; Table S4: Detailed information with stability analysis of the 29 novel SNPs identified in this study.

## References

[1] Lu R., Zhao X., Li J., Niu P., Yang B., Wu H., Wang W., Song H., Huang B., Zhu N. (2020). Genomic Characterisation and Epidemiology of 2019 Novel Coronavirus: Implications for Virus Origins and Receptor Binding. Lancet.

[2] Pedersen S.F., Ho Y.-C. (2020). SARS-CoV-2: A Storm Is Raging. J. Clin. Investig..

[3] Misra G., Manzoor A., Chopra M., Upadhyay A., Katiyar A., Bhushan B., Anvikar A. (2023). Genomic Epidemiology of SARS-CoV-2 from Uttar Pradesh, India. Sci. Rep..

[4] COVID-19 Cases | WHO COVID-19 Dashboard. https://data.who.int/dashboards/covid19/cases?n=c.

[5] Zaki A.M., van Boheemen S., Bestebroer T.M., Osterhaus A.D., Fouchier R.A. (2012). Isolation of a Novel Coronavirus from a Man with Pneumonia in Saudi Arabia. N. Engl. J. Med..

[6] Andrews M.A., Areekal B., Rajesh K.R., Krishnan J., Suryakala R., Krishnan B., Muraly C.P., Santhosh P.V. (2020). First Confirmed Case of COVID-19 Infection in India: A Case Report. Indian J. Med. Res..

[7] Singh S., Sharma A., Gupta A., Joshi M., Aggarwal A., Soni N., Sana, Jain D., Verma P., Khandelwal D. (2022). Demographic Comparison of the First, Second and Third Waves of COVID-19 in a Tertiary Care Hospital at Jaipur, India. Lung India.

[8] India COVID—Coronavirus Statistics—Worldometer. https://www.worldometers.info/coronavirus/country/india/.

[9] Pattabiraman C., Habib F., Harsha P.K., Rasheed R., Prasad P., Reddy V., Dinesh P., Damodar T., Hosallimath K., George A.K. (2020). Genomic Epidemiology Reveals Multiple Introductions and Spread of SARS-CoV-2 in the Indian State of Karnataka. PLoS ONE.

[10] Pattabiraman C., Desai A., Prasad P., George A.K., Sreenivas D., Rasheed R., Reddy N.V.K., Vasanthapuram R. (2021). Importation, Circulation, and Emergence of Variants of SARS-CoV-2 in the South Indian State of Karnataka. Wellcome Open Res.

[11] Singh B., Avula K., Chatterjee S., Datey A., Ghosh A., De S., Keshry S.S., Ghosh S., Suryawanshi A.R., Dash R. (2022). Isolation and Characterization of Five Severe Acute Respiratory Syndrome Coronavirus 2 Strains of Different Clades and Lineages Circulating in Eastern India. Front. Microbiol..

[12] Radhakrishnan C., Divakar M.K., Jain A., Viswanathan P., Bhoyar R.C., Jolly B., Imran M., Sharma D., Rophina M., Ranjan G. (2021). Initial Insights into the Genetic Epidemiology of SARS-CoV-2 Isolates from Kerala Suggest Local Spread from Limited Introductions. Front. Genet..

[13] Joshi M., Puvar A., Kumar D., Ansari A., Pandya M., Raval J., Patel Z., Trivedi P., Gandhi M., Pandya L. (2021). Genomic Variations in SARS-CoV-2 Genomes From Gujarat: Underlying Role of Variants in Disease Epidemiology. Front. Genet..

[14] Limaye S., Kasibhatla S.M., Ramtirthkar M., Kinikar M., Kale M.M., Kulkarni-Kale U. (2021). Circulation and Evolution of SARS-CoV-2 in India: Let the Data Speak. Viruses.

[15] Gupta A., Sabarinathan R., Bala P., Donipadi V., Vashisht D., Katika M.R., Kandakatla M., Mitra D., Dalal A., Bashyam M.D. (2021). A Comprehensive Profile of Genomic Variations in the SARS-CoV-2 Isolates from the State of Telangana, India. J. Gen. Virol..

[16] MoHFW | Home. https://www.mohfw.gov.in/#latest-update.

[17] Andrew S. (2010). FastQC. A Quality Control Tool for High Throughput Sequence Data. https://www.bioinformatics.babraham.ac.uk/projects/fastqc/.

[18] Bolger A.M., Lohse M., Usadel B. (2014). Trimmomatic: A Flexible Trimmer for Illumina Sequence Data. Bioinformatics.

[19] Li H. (2013). Aligning Sequence Reads, Clone Sequences and Assembly Contigs with BWA-MEM. arXiv.

[20] Danecek P., Bonfield J.K., Liddle J., Marshall J., Ohan V., Pollard M.O., Whitwham A., Keane T., McCarthy S.A., Davies R.M. (2021). Twelve Years of SAMtools and BCFtools. Gigascience.

[21] Aksamentov I., Roemer C., Hodcroft E.B., Neher R.A. (2021). Nextclade: Clade Assignment, Mutation Calling and Quality Control for Viral Genomes. J. Open Source Softw..

[22] Chen C.W., Lin M.H., Liao C.C., Chang H.P., Chu Y.W. (2020). IStable 2.0: Predicting Protein Thermal Stability Changes by Integrating Various Characteristic Modules. Comput. Struct. Biotechnol. J..

[23] Hadfield J., Megill C., Bell S.M. (2020). Genomic Epidemiology of Novel Coronavirus: Global Subsampling. Nextstrain: Real-Time Tracking of Pathogen Evolution.

[24] Hadfield J., Megill C., Bell S.M., Huddleston J., Potter B., Callender C., Sagulenko P., Bedford T., Neher R.A. (2018). Nextstrain: Real-Time Tracking of Pathogen Evolution. Bioinformatics.

[25] Minh B.Q., Schmidt H.A., Chernomor O., Schrempf D., Woodhams M.D., Von Haeseler A., Lanfear R. (2020). IQ-TREE 2: New Models and Efficient Methods for Phylogenetic Inference in the Genomic Era. Mol. Biol. Evol..

[26] Kumar S., Stecher G., Suleski M., Blair Hedges S. (2017). TimeTree: A Resource for Timelines, Timetrees, and Divergence Times. Mol. Biol. Evol..

[27] Letunic I., Bork P. (2021). Interactive Tree Of Life (ITOL) v5: An Online Tool for Phylogenetic Tree Display and Annotation. Nucleic Acids Res..

[28] Brown K.A., Gubbay J., Buchan S.A., Daneman N., Mishra S., Patel S., Day T. (2021). Inflection in Prevalence of SARS-CoV-2 Infections Missing the N501Y Mutation as a Marker of Rapid Delta (B.1.617.2) Lineage Expansion in Ontario, Canada. medRxiv.

[29] Mohapatra R.K., Tiwari R., Sarangi A.K., Sharma S.K., Khandia R., Saikumar G., Dhama K. (2022). Twin Combination of Omicron and Delta Variants Triggering a Tsunami Wave of Ever High Surges in COVID-19 Cases: A Challenging Global Threat with a Special Focus on the Indian Subcontinent. J. Med. Virol..

[30] Ray S.K., Mukherjee S. (2022). Divulging Incipient SARS-CoV-2 Delta (B.1.617.2) Variant: Possible with Global Scenario. Infect. Disord. Drug Targets.

[31] Jackson C.B., Zhang L., Farzan M., Choe H. (2021). Functional Importance of the D614G Mutation in the SARS-CoV-2 Spike Protein. Biochem. Biophys. Res. Commun..

[32] Khatri R., Siddqui G., Sadhu S., Maithil V., Vishwakarma P., Lohiya B., Goswami A., Ahmed S., Awasthi A., Samal S. (2023). Intrinsic D614G and P681R/H Mutations in SARS-CoV-2 VoCs Alpha, Delta, Omicron and Viruses with D614G plus Key Signature Mutations in Spike Protein Alters Fusogenicity and Infectivity. Med. Microbiol. Immunol..

[33] Franceschi V.B., Caldana G.D., de Menezes Mayer A., Cybis G.B., Neves C.A.M., Ferrareze P.A.G., Demoliner M., de Almeida P.R., Gularte J.S., Hansen A.W. (2021). Genomic Epidemiology of SARS-CoV-2 in Esteio, Rio Grande Do Sul, Brazil. BMC Genom..

[34] Ghosh A., Walia S., Rattan R., Kanampalliwar A., Jha A., Aggarwal S., Fatma S., Das N., Chayani N., Prasad P. (2022). Genomic Profiles of Vaccine Breakthrough SARS-CoV-2 Strains from Odisha, India. Int. J. Infect. Dis..

[35] Ferreira I.A.T.M., Kemp S.A., Datir R., Saito A., Meng B., Rakshit P., Takaori-Kondo A., Kosugi Y., Uriu K., Kimura I. (2021). SARS-CoV-2 B.1.617 Mutations L452R and E484Q Are Not Synergistic for Antibody Evasion. J Infect Dis.

[36] Pathak A.K., Mishra G.P., Uppili B., Walia S., Fatihi S., Abbas T., Banu S., Ghosh A., Kanampalliwar A., Jha A. (2022). Spatio-Temporal Dynamics of Intra-Host Variability in SARS-CoV-2 Genomes. Nucleic Acids Res.

[37] Rochman N.D., Wolf Y.I., Faure G., Mutz P., Zhang F., Koonin E.V. (2021). Ongoing Global and Regional Adaptive Evolution of SARS-CoV-2. Proc. Natl. Acad. Sci. USA.

[38] Gupta A.M., Chakrabarti J., Mandal S. (2020). Non-Synonymous Mutations of SARS-CoV-2 Leads Epitope Loss and Segregates Its Variants. Microbes Infect..

[39] Markov P.V., Ghafari M., Beer M., Lythgoe K., Simmonds P., Stilianakis N.I., Katzourakis A. (2023). The Evolution of SARS-CoV-2. Nat. Rev. Microbiol..

[40] Otto S.P., Day T., Arino J., Colijn C., Dushoff J., Li M., Mechai S., Van Domselaar G., Wu J., Earn D.J.D. (2021). The Origins and Potential Future of SARS-CoV-2 Variants of Concern in the Evolving COVID-19 Pandemic. Curr. Biol..

[41] Bano I., Sharif M., Alam S. (2022). Genetic Drift in the Genome of SARS-CoV-2 and Its Global Health Concern. J. Med. Virol..

