# Supplementary figures and images for: Molecular Characterization and Genomic Surveillance of SARS-CoV-2 Lineages in Central India
Source: Viruses. 2024 Oct 14;16(10):1608. doi: 10.3390/v16101608 (PMC11512289; doi:10.3390/v16101608)

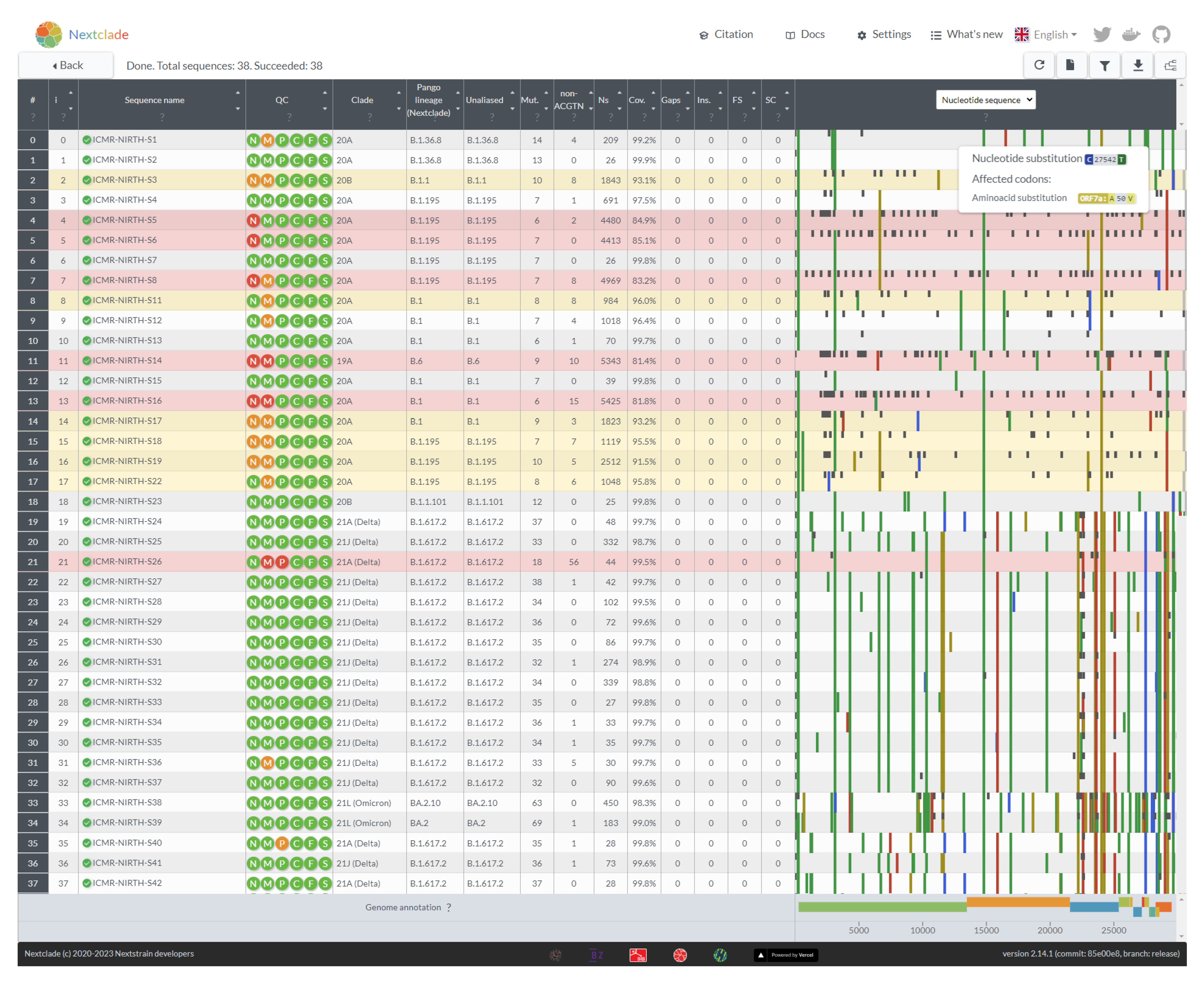

Supplement: Supplementary file 1 [file viruses-16-01608-s001.zip › Supplementary Figure_S1.tif]
